# Development and internal validation of the multivariable CIPHER (Collaborative Integrated Pregnancy High-dependency Estimate of Risk) clinical risk prediction model

**DOI:** 10.1186/s13054-018-2215-6

**Published:** 2018-10-30

**Authors:** Beth A. Payne, Helen Ryan, Jeffrey Bone, Laura A. Magee, Alice B. Aarvold, J. Mark Ansermino, Zulfiqar A. Bhutta, Mary Bowen, J. Guilherme Cecatti, Cynthia Chazotte, Tim Crozier, Anne-Cornélie J. M. de Pont, Oktay Demirkiran, Tao Duan, Marlot Kallen, Wessel Ganzevoort, Michael Geary, Dena Goffman, Jennifer A. Hutcheon, K. S. Joseph, Stephen E. Lapinsky, Isam Lataifeh, Jing Li, Sarka Liskonova, Emily M. Hamel, Fionnuala M. McAuliffe, Colm O’Herlihy, Ben W. J. Mol, P. Gareth R. Seaward, Ramzy Tadros, Turkan Togal, Rahat Qureshi, U. Vivian Ukah, Daniela Vasquez, Euan Wallace, Paul Yong, Vivian Zhou, Keith R. Walley, Peter von Dadelszen, the CIPHER Group, Joni Kooy, Brittany Tarras, Nancy Liu, Rebecca Gordon, Shannon Lockhart, Annie Tran, Run Shan Felar Yu, Yisa Yen, Andy Dhaliwal, Chris Lim, Nelson Luk, Saba Marzara, Navdeep Dha, Niamh Barrett, Lucia Haritgan, Evan Lambe, Aoife Doyle, Aisling McMahon, Richard Katz, Andrea Das Neves, Vanina Aphalo, Marlot Kallen, Colleen Lee, Katey Austin, Mary Mahler, Dinusha Sen, Alina Blazer, Xiaotian Ni, Sheikh Irfan, Azra Amerjee, Antonio F. Oliveira Neto, Mary Angela Parpinelli, Maria Laura Costa, Thais Giovarotti, Etienne Cordeiro

**Affiliations:** 1 Department of Obstetrics and Gynaecology, University of British Columbia, 2329 West Mall, Vancouver, V6T 1Z4 BC Canada; 2 Department of Anesthesiology, Pharmacology and Therapeutics, University of British Columbia, 2329 West Mall, Vancouver, V6T 1Z4 BC Canada; 3 BC Children’s Hospital Research Institute and Women’s Health Research Institute, University of British Columbia, V3-336 950 W 28th Avenue, Vancouver, BC V5Z 4H4 Canada; 4 Department of Family Practice, University of British Columbia, 2329 West Mall, Vancouver, V6T 1Z4 BC Canada; 5 Department of Medicine, University of British Columbia, 2329 West Mall, Vancouver, V6T 1Z4 BC Canada; 6 School of Life Course Sciences, Faculty of Life Sciences and Medicine, King’s College London, Strand, London, WC2R2LS UK; 7 Center of Excellence in Women & Child Health, Aga Khan University, Block 2 Clifton, Karachi, Sindh Pakistan; 8 Centre for Global Child Health, the Hospital for Sick Children, 555 University Ave, Toronto, M5G 1X8 ON Canada; 9 Rotunda Hospital, University College Dublin, 1, Parnell Square E, Dublin, Ireland; 10 Universidade Estadual de Campinas, Cidade Universitaria Zeferino Vaz - Barao Geraldo, Campinas, 13083-970 Sao Paulo Brazil; 11 Montefiore Medical Center, Columbia University Medical Center, 951 Prospect Ave, Bronx, 10459 NY USA; 12 Morgan Stanley Children’s Hospital & Sloan Hospital for Mothers, 10032, 3959 Broadway, New York, NY USA; 13 Department of Obstetrics and Gynaecology, Monash University, Wellington Rd, Clayton, 3800 Victoria Australia; 14 Academic Medical Centre, Meibergdreef 9, Amsterdam, 1105 AZ The Netherlands; 15 Inonu University, Bulgurlu Mahallesi, Malatya, 44000 Battalgazi Turkey; 16 Shanghai 1st Maternity and Infant Hospital, 200000, 536 Changle Rd, Shanghai, Jingan Qu China; 17 Mt Sinai Hospital, University of Toronto, 600 University Ave, Toronto, M5G1X5 ON Canada; 18 King Abdullah University Hospital, Ar Ramtha, 3030 Ramtha Jordan; 19 UCD Perinatal Research Centre, School of Medicine, University College Dublin, National Maternity Hospital, Belfield Downs, Dublin, D14YH57 Ireland; 20 Department of Paediatrics and Women’s Health, University of Adelaide, Adelaide, 5005 SA Australia; 21 Hospital Interzonal General de Agudos Gral, Av. 101 Dr Ricardo Balbin, Buenos Aires, 3200 Argentina; 22 Centre for Heart Lung Innovation, St Paul’s Hospital, 1081 Burrard Street, Vancouver, V6Z1Y6 BC Canada

**Keywords:** Risk prediction model, High-risk pregnancy, Maternal mortality, Maternal morbidity, Critical care

## Abstract

**Background:**

Intensive care unit (ICU) outcome prediction models, such as Acute Physiology And Chronic Health Evaluation (APACHE), were designed in general critical care populations and their use in obstetric populations is contentious. The aim of the CIPHER (Collaborative Integrated Pregnancy High-dependency Estimate of Risk) study was to develop and internally validate a multivariable prognostic model calibrated specifically for pregnant or recently delivered women admitted for critical care.

**Methods:**

A retrospective observational cohort was created for this study from 13 tertiary facilities across five high-income and six low- or middle-income countries. Women admitted to an ICU for more than 24 h during pregnancy or less than 6 weeks post-partum from 2000 to 2012 were included in the cohort. A composite primary outcome was defined as maternal death or need for organ support for more than 7 days or acute life-saving intervention. Model development involved selection of candidate predictor variables based on prior evidence of effect, availability across study sites, and use of LASSO (Least Absolute Shrinkage and Selection Operator) model building after multiple imputation using chained equations to address missing data for variable selection. The final model was estimated using multivariable logistic regression. Internal validation was completed using bootstrapping to correct for optimism in model performance measures of discrimination and calibration.

**Results:**

Overall, 127 out of 769 (16.5%) women experienced an adverse outcome. Predictors included in the final CIPHER model were maternal age, surgery in the preceding 24 h, systolic blood pressure, Glasgow Coma Scale score, serum sodium, serum potassium, activated partial thromboplastin time, arterial blood gas (ABG) pH, serum creatinine, and serum bilirubin. After internal validation, the model maintained excellent discrimination (area under the curve of the receiver operating characteristic (AUROC) 0.82, 95% confidence interval (CI) 0.81 to 0.84) and good calibration (slope of 0.92, 95% CI 0.91 to 0.92 and intercept of −0.11, 95% CI −0.13 to −0.08).

**Conclusions:**

The CIPHER model has the potential to be a pragmatic risk prediction tool. CIPHER can identify critically ill pregnant women at highest risk for adverse outcomes, inform counseling of patients about risk, and facilitate bench-marking of outcomes between centers by adjusting for baseline risk.

**Electronic supplementary material:**

The online version of this article (10.1186/s13054-018-2215-6) contains supplementary material, which is available to authorized users.

## Background

Most women who die during or soon after pregnancy in a health facility do so in an intensive care unit (ICU), if one is available [1–3]. Maternal ICU admissions result from both obstetric and non-obstetric complications in pregnancy; about two thirds of admissions are due to obstetric causes, such as hemorrhage, pre-eclampsia, and sepsis, and one third are due to maternal medical or surgical complications [1–3].

ICU clinical prediction models—including Acute Physiology And Chronic Health Evaluation II (APACHE II), APACHE III, Multiple Organ Dysfunction Score (MODS), Simplified Acute Physiology Score 3 (SAPS 3), and Sepsis-related Organ Failure Assessment (SOFA)—were developed in general ICU populations to assess the likelihood of an adverse health outcome (such as death or severe morbidity) and guide counseling and clinical decision making [4–8]. However, most ICU outcome prediction models were designed in general critical care populations in high-income countries (HICs) and their use in obstetric populations and in low- and middle-income countries (LMICs) is contentious. These general ICU prediction rules tend to overestimate the risk of maternal death by up to 20-fold [1]. The two exceptions to this are the SOFA score for sepsis risk and the Maternal Severity Index rule [9, 10]. None of the obstetric-focused ICU risk models has been developed to predict maternal death or prolonged organ support, an outcome reflective of severe maternal morbidity and of greater relevance in maternity populations among whom death is unusual, even in the ICU*.*

Maternal Early Warning scores are being adopted in obstetric care in many settings across the globe. These scores have resulted in mixed results when validated [11] and are relevant only for care prior to ICU admission. Given the unique physiology during pregnancy and post-partum, new pregnancy-specific clinical prediction rules, specific to the ICU setting, are required [12, 13]. Our objective was to develop and internally validate the globally relevant CIPHER (Collaborative Integrated Pregnancy High-dependency Estimate of Risk) model to predict either death or severe morbidity for pregnant and post-partum women admitted for critical care.

## Methods

### Study setting

Thirteen collaborating sites with ICUs from 11 countries contributed data to the CIPHER cohort. These sites were identified through a literature review of published obstetric ICU cohorts. After initial contact to establish whether the investigators were interested in collaborating, they were sent a survey to ensure suitability of the facility for participation in the CIPHER cohort. This survey evaluated human resources, interventions, and infrastructure available at each ICU site to ensure that sites were similar with regard to type of care provided. The HICs were in Canada, the US, Ireland, the Netherlands, and Australia. The LMIC sites were in Brazil, Argentina, Jordan, Turkey, Pakistan, and China. (For details, please see Additional file 1: Table S1.)

### Inclusion and exclusion criteria

Women were included if they were admitted to a critical care unit for more than 24 h and were known to be either pregnant (diagnosed before or during their ICU stay) or no more than 42 days post-partum, irrespective of pregnancy duration, from 1 January 2000 to 31 December 2012. Women admitted for less than 24 h who were neither pregnant nor recently pregnant were excluded, as were women with 10 or more missing candidate predictor variables or those who were missing primary outcome information or who met the definition of the primary outcome prior to admission.

### Data collection

We carried out a retrospective chart review of both paper and electronic medical records by using standardized data collection forms and protocols for all sites. Data were entered into a customized, secure online RedCAP^®^ database. All data were reviewed for quality and consistency. When questions arose regarding data reliability, these data were confirmed by re-review of the primary health record.

### Primary outcome

The composite primary outcome was defined as any one of (i) maternal death during pregnancy or within 42 days of delivery or (ii) organ support for more than 7 days or (iii) life-saving intervention or a combination of these. Organ support and life-saving interventions included in this composite were defined by organ system and are used as a surrogate for severe maternal morbidity. Specifically, organ support outcomes include any one or more of (1) respiratory (continuous positive airway pressure (CPAP), bilevel positive airway pressure (BiPAP), or invasive ventilation); (2) cardiac (positive inotrope or vasopressor use); (3) continuous renal replacement therapy for acute renal failure; (4) hepatic (liver transplantation and other management of hepatic failure (for example, ventilatory and circulatory support), management of elevated intracranial pressure and renal failure, and medical therapies for hepatitis B virus (for example, lamivudine)), and anticoagulation for Budd–Chiari syndrome); (5) hematologic (transfusion of at least 5 units of blood products); (6) neurological (Glasgow Coma Scale score of less than 10); or (7) uterine (uncontrollable hemorrhage or infection leading to hysterectomy). These definitions were arrived at through study working group consensus and are based on definitions of organ support used in the APACHE studies [5] and the World Health Organization (WHO) near-miss approach [10].

### Sample size

This sample size estimate is based on a rule of thumb for developing risk prediction models with unbiased estimates of regression coefficients [14]. The formula—[N = (n × 10) / I]—was used to calculate the sample size where N = the required sample size, n = the number of variables to be tested, and I = the incidence of the combined adverse outcome [14]. We assumed, on the basis of published reports, *I* = 12% for either maternal mortality or prolonged organ support for obstetric women admitted for critical care [15]. We estimated that to develop a reliable model with minimal overfitting with *n* = 10 candidate predictor variables at an assumed event rate of 12%, a cohort of *N* = 833 women was required.

### Model development

#### Dealing with missing data

Multiple imputation using chained eqs [16, 17] was undertaken to estimate missing data over 10 iterations to generate 10 complete datasets for model development. Two analysts replicated this process. We assumed that data were missing at random [17]. Women with and without missing data were compared to identify all clinical, laboratory, and demographic variables that differed between the groups. These variables were included in the imputation models along with all selected candidate predictor variables and the primary outcome [18].

#### Selection of candidate predictor variables

Initially, we performed a structured literature review of existing critical care outcome prediction models and their evidence for use in pregnancy to identify candidate predictor variables [1]. This review has been published elsewhere [1]. Variables considered for the CIPHER model included patient demographic details, prior health status, indication for ICU admission, and clinical and laboratory measurements taken in the first 24 h following ICU admission. The literature review identified 43 possible variables to include in the modelling process. This list was refined and reduced to 19 after exclusion of variables that were not routinely available at all sites (defined as having more than 30% missing values in the dataset) and through iterative dialogue with the participating critical care, maternal-fetal-medicine, and epidemiology experts in the CIPHER team to identify concerns about generalizability of measurements and clinical policy relevant to each variable across study settings.

**Fig. 1:**
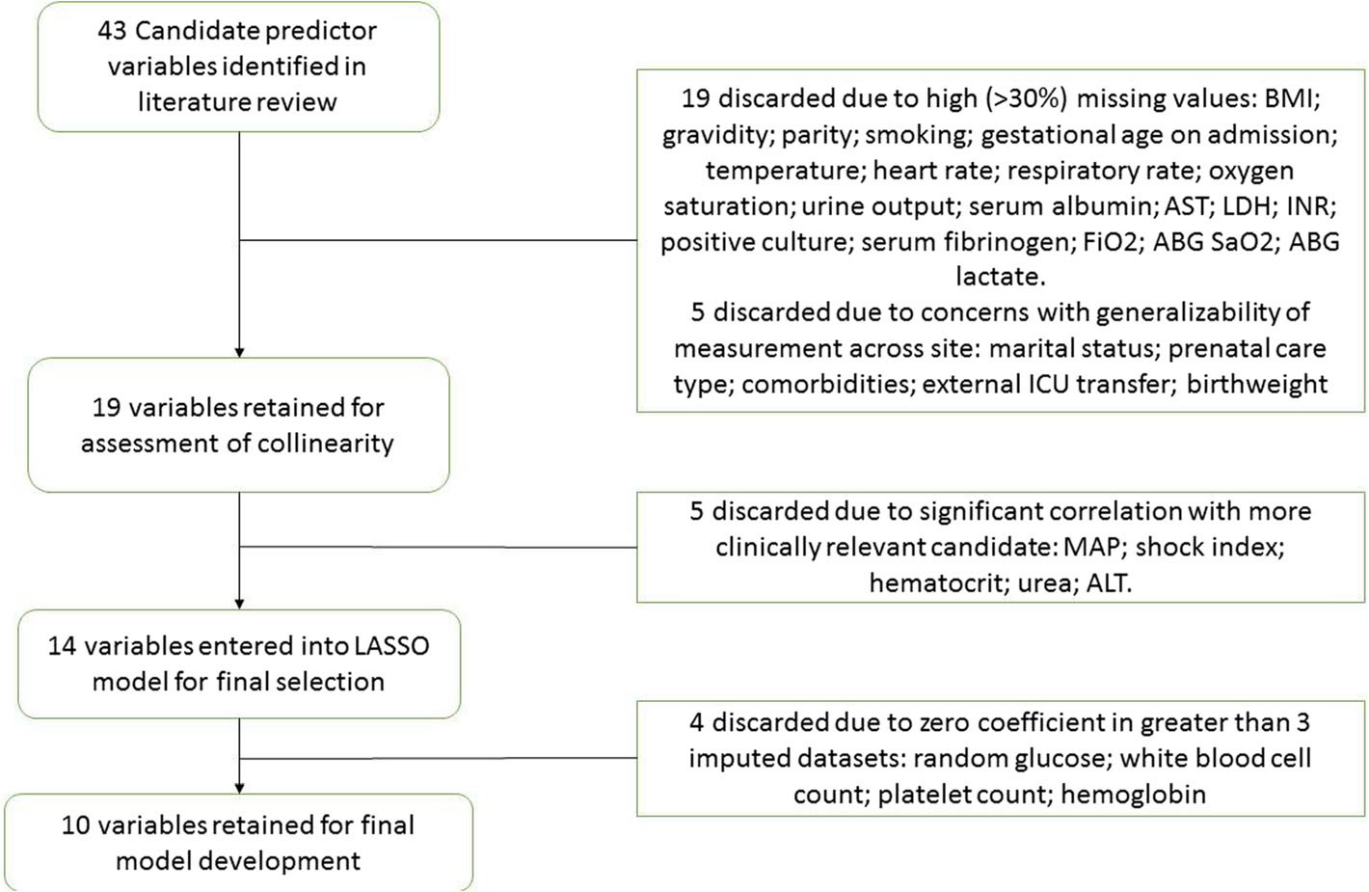
The process of selection of predictor variables for inclusion in the CIPHER (Collaborative Integrated Pregnancy High-dependency Estimate of Risk) model. Abbreviations: *ABG* arterial blood gas, *ALT* alanine aminotransferase, *AST* aspartate aminotransferase, *BMI* body mass index, *FiO2* fraction of inspired oxygen, *ICU* intensive care unit, *INR* international normalized ratio, *LASSO* Least Absolute Shrinkage and Selection Operator, *LDH* lactate dehydrogenase, *MAP* mean arterial pressure, *SaO2* oxygen saturation.

As a final step, Pearson’s correlation coefficient (for continuous predictors) or chi-squared test (for categorical variables) was used to estimate any correlation between candidate predictors within each imputed dataset. Consultation within the study working group was used when collinearity was suspected to select which variable to retain on the basis of perceived clinical value, reliability of measurement, and availability. The 14 most clinically relevant, available, and non-correlated variables were then included in the final variable selection step (Fig. 1).

The LASSO (Least Absolute Shrinkage and Selection Operator) method was used to reduce the number of candidate predictors further and select final variables to include in the CIPHER model [19]. The LASSO selects variables by penalizing models that have more and larger coefficients. This requires a choice for the degree to which to penalize these terms. For this analysis, the penalty term in each imputed dataset was chosen by 10-fold cross-validation. This process resulted in a variety of possible penalty “sizes”, and the one in each dataset yielding the smallest area under the curve of the receiver operating characteristic (AUROC) was chosen as the penalty to use. Both squared and cubic transformations were evaluated at this stage of variable selection for any candidate predictor variable that was assumed to have a non-linear relationship with the outcome due to known increases in clinical risk at both high and low levels. These variables were identified prior to LASSO model development as maternal age, blood pressure, serum sodium, and white blood cell count.

LASSO models were built using the glmnet package in R [20]. This variable selection technique was repeated within each imputed dataset, resulting in 10 models. *A priori*, we set thresholds for inclusion in the final CIPHER model as any variable which retained a non-zero coefficient in the LASSO model in seven or more imputations. Variables that maintained a non-zero coefficient in fewer than seven of the LASSO models would not be retained for final parameter estimation.

#### Estimating parameter effects

Multivariable logistic regression was used to estimate variable effects of selected predictors in each of the 10 imputed datasets. A final pooled estimate of effect was then generated using Rubin’s rules implemented in STATA through the mi estimate command [21].

#### Assessing the model’s performance

The discrimination ability of the final model was evaluated on the basis of AUROC [22]. Discrimination in this context refers to the ability of the model to distinguish between women with and without outcomes. An AUROC of more than 0.7 indicates good model discrimination.

Model calibration was assessed by plotting deciles of the predicted probability of an adverse maternal outcome against the observed rate in each decile and fitting a smooth line using locally weighted scatterplot smoothing (lowess) using the “rms” and “calibrationcurves” packages in R [23, 24]. This smooth line is used to determine the calibration slope and intercept [25]. These calibration measures are used to describe the accuracy of the predicted probability compared with the observed outcome and are considered measures of model goodness-of-fit. Ideally, the slope would be close to one and intercept zero. Discrimination and calibration measures were estimated for each of the imputed datasets and then pooled using Rubin’s rules. Graphs presented are drawn using the pooled linear predictor value for each woman in the dataset.

A risk stratification table was used to evaluate model classification accuracy and stratification capacity. Both classification accuracy, defined as the ability of the model to separate cases with an outcome into higher-risk groups and cases without an outcome into lower-risk groups, and stratification capacity, defined as the ability of the model to separate the population into distinct risk groups, give additional information on model calibration. A useful model will separate the population into distinct risk groups so that the majority of outcome cases result in a high predicted probability and the remaining cases have a visibly lower predicted probability, leaving few in the middle.

Categories within the stratification table were defined to be balanced around the population prevalence. By balancing around the population prevalence, we defined risk groups that have the potential to be meaningfully different than the prevalence itself, which represents the total population risk. Specifically, the lowest and highest groups were set at predictive probability about three times greater and less than the prevalence. We calculated the sensitivity, specificity, positive predictive value, negative predictive value, and likelihood ratios (LRs) for each risk group. A positive test was defined using the upper limit of the predicted probability range for each risk group, except for calculation of the LRs, which followed the method of Deeks and Altman [26]. These measures of diagnostic accuracy are used in this study to describe potential accuracy of the model if it were implemented as a decision rule using the defined risk groups. The following categories for interpretation of the LRs were used: strongly informative (LR <0.1 or >10), moderately informative (LR 0.1–0.2 or 5–10), and non-informative (LR 0.2–5). Uniformity of the model fit was tested by assessing model performance in various subsets of study data, including HIC versus LMIC, antenatal versus post-partum admission, and in cases with obstetric versus other indication for admission.

#### Model internal validation and optimism correction

Internal validation of the model was assessed in each of the 10 imputed datasets using Efron’s enhanced bootstrap method [27]. Details of this approach have been described previously [28, 29]. Model optimism was calculated for discrimination (AUROC) as the average difference between model performance in the bootstrap sample and the original imputed dataset after 200 iterations of the bootstrap procedure. This resulted in 10 estimates of average optimism, which were pooled using Rubin’s rules to generate a final optimism result.

In addition, internal validation of the calibration slope and intercept was completed during the bootstrapping procedure using the same method as above. For these measures of model fit, we calculated the average slope and intercept for each bootstrap model applied to its original imputed dataset over 200 iterations. This resulted in 10 average slopes and intercepts that were then pooled using Rubin’s rules to generate final internally validated measures of model calibration.

All analyses were initially performed in R using the “mice”, “rms”, “calibrationcurves”, and “glmnet” packages. Pooling of parameter estimates and model performance estimates were repeated by a second analyst to confirm results using STATA version 13.0.

## Results

At the 13 study sites, retrospective chart review was completed for 876 eligible women who met inclusion criteria. We excluded 107 women; 93 of these were excluded because they were missing at least 10 out of 19 candidate predictor variables included in the imputation step and 14 because they were missing outcome data. A final cohort of 769 women was identified for analysis.

Characteristics of the study population are presented in Table 1, comparing women with and without the primary adverse outcome of death or morbidity, as previously defined. Women with the primary adverse outcome were younger and more often accessed care through private facilities, were admitted for non-obstetric reasons, were admitted to the ICU earlier in gestation and for longer, and had a greater number of early pregnancy losses and stillbirths. The primary outcome was observed in 127 (16.5%) women. Of these, 59 (7.7%) were maternal deaths and 68 (8.8%) required one or more component of organ support alone. The most common organ support outcome was the need for respiratory support (Table 2).

**Table 1:** Characteristics of the study population

Patient characteristics	Women with no outcome (*n* = 642), median (IQR) or n (%)	Women with outcome (*n* = 127), median (IQR) or n (%)
Age, years	31 (26–36)	27 (25–32)
Marital status		
Married	346 (53.9)	90 (70.9)
Single	62 (9.7)	1 (0.8)
Missing	234 (36.4)	36 (28.3)
BMI, kg/m^2^	26.4 (24.0–30.0)	26.2 (23.2–29.1)
Prenatal care type		
Public	383 (59.7)	54 (42.5)
Private	208 (32.4)	73 (57.5)
Missing	51 (7.9)	0 (0.0)
Gravidity	3 (2–4)	4 (2–5)
Parity	1 (1–2)	2 (1–3)
Details of ICU admission		
Reason for admission‡		
Obstetric*	465 (72.4)	77 (60.6)
Non-obstetric**	263 (41.0)	95 (74.8)
Missing	3 (0.4)	0 (0.0%)
Timing of admission		
Antepartum	162 (25.2)	46 (36.2)
Intrapartum	6 (0.9)	1 (0.8)
Post-partum	459 (71.5)	77 (60.6)
Missing	15 (2.3)	3 (2.4)
Gestational age in weeks at ICU admission (for women admitted antepartum)	34.4 (28.7–38.0)	30.9 (22.4–35.8)
External ICU transfer, yes	184 (28.7)	21 (16.5)
Length of ICU stay, hours	59.4 (37.0–92.1)	209.9 (110.7–309.4)
Pregnancy outcomes		
Early pregnancy loss <24 weeks		
Yes	59 (9.2)	27 (21.3)
Missing	29 (4.5)	13 (10.2)
Stillbirth	33 (5.1)	24 (18.9)
Livebirth	459 (71.5)	55 (43.3)
Mode of birth		
Vaginal	96 (19.8)	25 (30.5)
Caesarean	387 (79.8)	57 (69.5)
Missing	2 (0.4)	0 (0.0)
Birth weight, g	2555 (1700–3250)	2100 (1415–2700)

Abbreviations: *BMI* body mass index, *ICU* intensive care unit, *IQR* interquartile range

*Obstetric reasons for admission included shock, massive postpartum hemorrhage, peripartum cardiomyopathy, amniotic fluid embolism, acute respiratory distress secondary to antepartum hemorrhage, pulmonary edema secondary to pre-eclampsia, eclampsia, septic abortion, other septic complications, and surgical trauma.

**Non-obstetric reasons for admission included cardiac arrhythmia, pericardial effusions, cardiogenic pulmonary edema, pulmonary hypertension, cardiac arrest, pneumonia, respiratory failure or arrest, gastrointestinal perforation/obstruction, diabetic keto-acidosis, deep venous thrombosis, thrombotic thrombocytopenic purpura, posterior reversible encephalopathy syndrome, and severe infection with sepsis

‡ reasons are not mutually exclusive

**Table 2:** Incidence and definition of each of the components of the primary outcome

	Definition	Total (*n* = 769), *n* (%)
Total women with primary outcome		127 (16.5%)
Maternal death	Death during or within 42 days of delivery	59 (7.7%)
Maternal Morbidity	Occurrence of one or more of the defined organ support measures below	97 (12.6%)
Organ support		
Respiratory	Need for CPAP, BiPAP, or invasive ventilation	73
Cardiovascular	Need for use of inotropes or vasopressors	15
Renal	Renal replacement therapy for acute renal failure	14
Hepatic	Liver transplantation. Other management of hepatic failure include ventilatory and circulatory support, management of elevated intracranial pressure and renal failure, medical therapies for hepatitis B virus (lamivudine); anticoagulation for Budd–Chiari syndrome.	21
Hematological	Massive transfusion of at least 5 units of blood products	60
Neurological	GCS score of less than 10	35
Uterine	Uncontrollable hemorrhage or infection leading to life-saving hysterectomy	16

Abbreviations: *BiPAP* bilevel positive airway pressure, *CPAP* continuous positive airway pressure, *GCS* Glasgow Coma Scale

Maternal morbidities presented are not mutually exclusive and include those occurring in women who died. Morbidities have been grouped by organ system. All organ system outcomes required use of life-saving treatment for more than 7 days to meet outcome criteria with the exception of hematological and uterine support.

### Imputation

A comparison of women with and without missing data shows that women with missing data were less severely ill and had shorter duration of ICU stay (Additional file 1: Table S3). Imputation models specified for this study included all variables that differed between women with or without one or more missing selected predictor variable to best account for these differences. Univariate odds ratios estimated for all candidate predictor variables in both the imputed data (Table 3) and complete case cohorts (Additional file 1: Table S2) were similar, and an expected increase in precision was seen in the estimates generated using the imputed data.

**Table 3:** Univariate and multivariable odds ratios for selected candidate predictor variables pooled from 10 imputed datasets

Patient characteristics multivariate analysis	Univariate OR (95% CI)	Multivariate OR (95% CI)
Maternal age, years	0.95 (0.92–0.98)	0.95 (0.92–0.99)
Surgery in preceding 24 h, yes	0.47 (0.32–0.70)	0.46 (0.29–0.73)
Highest systolic blood pressure, mm Hg	0.99 (0.98–1.02)	0.99 (0.98–1.00)
Lowest Glasgow Coma Scale score	0.85 (0.82–0.89)	0.87 (0.83–0.91)
Lowest ABG pH	0.60 (0.28–1.29)	0.57 (0.22–1.44)
Highest aPTT	1.02 (1.02–1.03)	1.02 (1.01–1.03)
Highest serum potassium	0.90 (0.72–1.12)	0.73 (0.56–0.94)
Highest serum sodium	1.07 (1.04–1.10)	1.03 (1.00–1.08)
Highest creatinine, per 10 unit change	1.05 (1.03–1.07)	1.04 (1.03–1.07)
Highest bilirubin, per 10 unit change	1.10 (1.07–1.14)	1.05 (1.01–1.09)

Abbreviations: *ABG* arterial blood gas, *aPTT* activated partial thromboplastin time, *CI* confidence interval, *OR* odds ratio

**Table 4:** Final CIPHER (Collaborative Integrated Pregnancy High-dependency Estimate of Risk) model

Logit(p) = 3.087 + [−1.912 × 10^−5^ (maternal age)^3^] + [−0.776(positive surgical status within 24 h of admission)] + [−0.138 (Glasgow Coma Scale score)] + [−7.123 × 10^−3^ (systolic blood pressure)] + [−0.319 (serum potassium)] + [1.373 × 10^−4^ (serum sodium)^2^] + [4.934 × 10^−3^ (serum bilirubin)] + [4.673 × 10^−3^ (serum creatinine)] + [1.584 × 10^−2^ (activated partial thromboplastin time)] + [−0.570 (arterial blood gas pH)] Maternal age (years); surgical status (yes/no); Glasgow Coma Scale score (ordinal units); systolic blood pressure (millimeter of mercury); serum bilirubin (micromole per liter); serum creatinine (micromole per liter)	

### Model development

The final variables selected for inclusion in the CIPHER model were maternal age, surgical status in the preceding 24 h, systolic blood pressure, Glasgow Coma Scale score, serum sodium, serum potassium, activated partial thromboplastin time (aPTT), serum creatinine, and serum bilirubin, and arterial blood gas (ABG) pH (Table 4). The odds of experiencing an adverse outcome increase as serum creatinine, total bilirubin, serum sodium, and aPTT increase; decrease as maternal age and systolic blood pressure decrease; and decrease if ABG pH and Glasgow Coma Scale score increase or if there was surgery in the 24 h preceding ICU admission (Table 3).

### Model performance

**Fig. 2:**
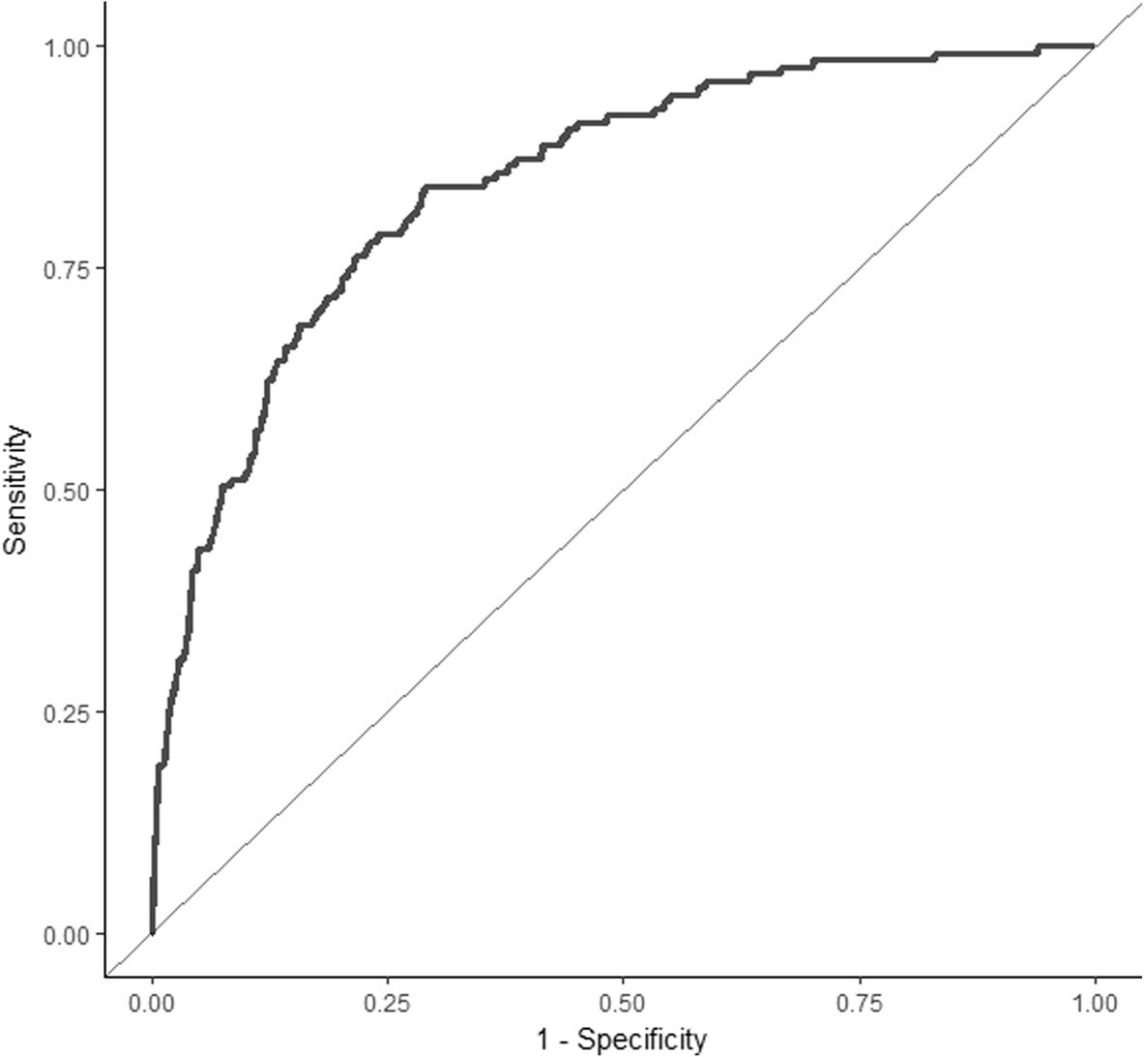
Area under the receiver operating characteristic curve for the CIPHER (Collaborative Integrated Pregnancy High-dependency Estimate of Risk) model plotted using the pooled predicted probabilities of outcome for each woman in the 10 imputed datasets. The area under the curve of the receiver operating characteristic for this model prior to adjustment for overfitting is 0.84 (0.83–0.85)

**Fig. 3:**
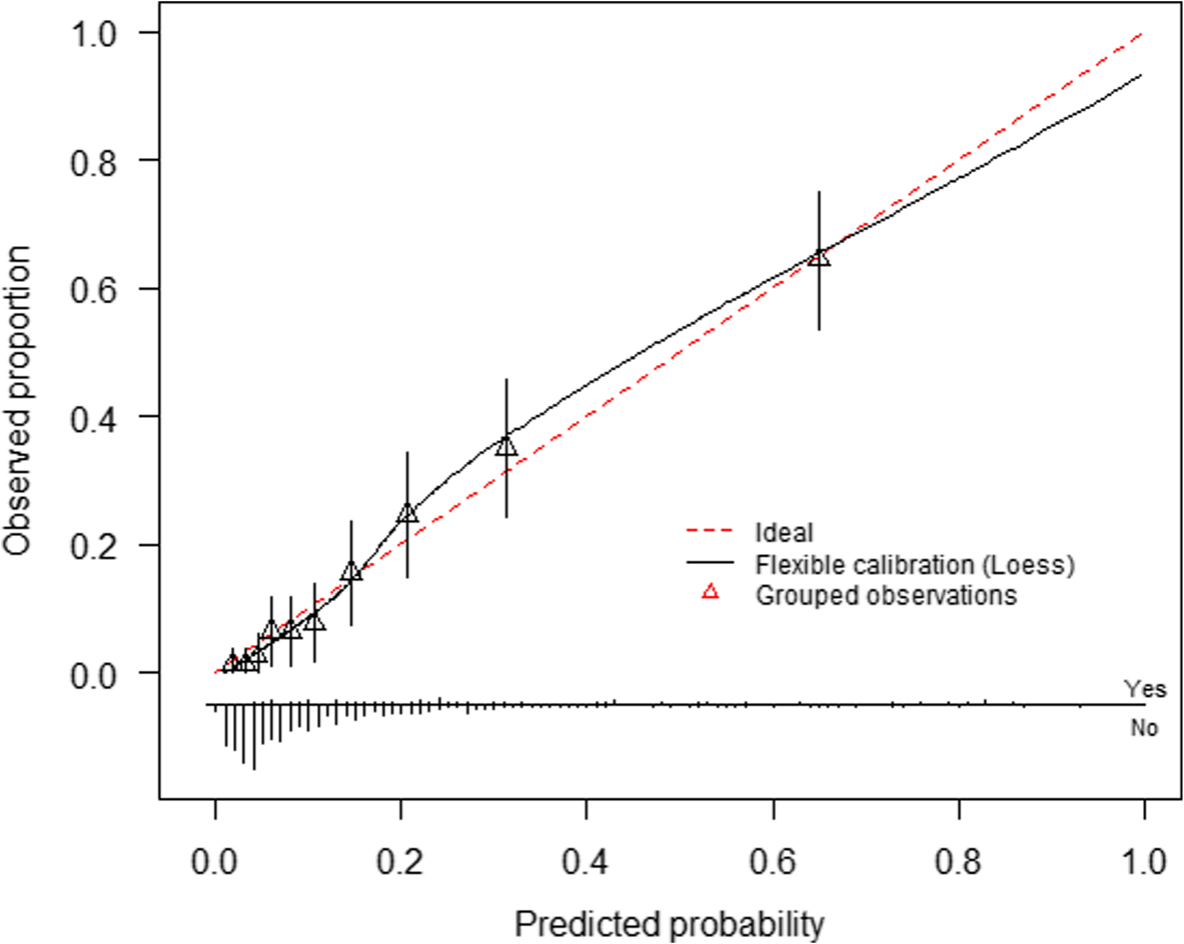
Calibration plot of CIPHER (Collaborative Integrated Pregnancy High-dependency Estimate of Risk) model developed using the pooled predicted probabilities of outcome for each woman in the 10 imputed datasets. The smooth line represents fit of the model predicted risk of outcome to the observed rate within each decile of predicted probability. The straight diagonal line is used as reference for perfect fit. The bar chart at the base of the figure presents distribution of cases with outcomes (above the line) and without cases (below the line) across the spectrum of predicted probability

The apparent AUROC for the CIPHER model was 0.84 (0.83 to 0.85) (Fig. 2). This model was well calibrated in the development data, as would be expected, with a calibration slope of 1.0 and intercept of −0.001 (Fig. 3). Stratification capacity and classification accuracy of the model as presented in Table 5 were both good; the majority of women identified in the two lowest risk groups (54.1% women, *n* = 416) had low rates of adverse outcome (3.8%, *n* = 16). Women in the highest-risk group had a high incidence of adverse maternal outcome (66.1%, *n* = 59). This is meaningfully greater than the population prevalence of adverse outcome (16.5%), as is reflected by the high LR associated with this category (27.97, 95% confidence interval (CI) 16.91 to 46.27).

**Table 5:** Risk stratification table using five groups of predicted probability

Predicted probability range	Women in range, n (%)	Observed outcome in range, n (%)	Sensitivity (95% CI)	Specificity (95% CI)	PPV (95% CI)	NPV (95% CI)	LR (95% CI)
<5%	236 (30.7)	5 (2.1)	–	–	–	–	0.31 (0.13–0.77)
5–9.9%	180 (23.4)	11 (8.7)	96.1 (90.6–98.5)	36.0 (32.2–39.8)	22.9 (19.4–26.7)	97.9 (94.8–99.2)	0.93 (0.52–1.67)
10–24.9%	212 (27.6)	40 (18.9)	87.4 (80.0–92.4)	62.3 (58.4–66.0)	31.4 (26.7–36.6)	96.2 (93.7–97.7)	3.34 (2.51–4.34)
25–49.9%	82 (10.7)	32 (39.0)	55.9 (46.8–64.6)	89.1 (86.4–91.3)	50.4 (41.9–58.8)	91.1 (88.5–93.1)	9.18 (6.16–13.69)
≥50%	59 (7.7)	39 (66.1)	30.7 (23.0–39.6)	96.9 (95.1–98.0)	66.1 (52.5–77.6)	87.6 (84.9–89.9)	27.97 (16.91–46.27)
Total	769	127					

Sensitivity, specificity, and predictive values calculated using the upper limit of the risk range to define a positive test.

Abbreviations: *CI* confidence interval, *LR* likelihood ratio, *NPV* negative predictive value, *PPV* positive predictive value

### Internal validation

After 200 iterations of bootstrapping in each of the 10 imputed datasets, the pooled average optimism for the AUROC was 0.013, which results in an optimism-corrected AUROC for the CIPHER model of 0.82 (95% CI 0.81 to 0.84). Minimal overestimation of risk was identified after bootstrap analysis with a resultant optimism-corrected calibration slope of 0.92 (95% CI 0.91 to 0.92) and intercept of −0.11 (95% CI −0.13 to −0.08).

### Subgroup analysis

All subgroup analyses demonstrated uniformity of model fit with maintenance of discriminative performance of the CIPHER model above our defined threshold for adequate performance of AUROC of more than 0.7. When the cohort was restricted to only those cases admitted to the ICU during the antenatal/intrapartum period, versus the post-partum period, the AUROCs were estimated as 0.84 (95% CI 0.78 to 0.91) and 0.83 (95% CI 0.79 to 0.89), respectively. When the cohort was restricted to only those cases admitted for obstetric causes versus those with non-obstetric causes, the AUROCs were 0.85 (95% CI 0.80 to 0.90) and 0.82 (95% CI 0.76 to 0.88), respectively. When the cohort was restricted to either LMIC facilities or HIC facilities, the AUROCs were estimated to be 0.851 (95% CI 0.812 to 0.894) versus 0.774 (95% CI 0.663 to 0.868), respectively. There was a small but meaningful decrease in the point estimate of AUROC when the analysis was restricted to only HIC cases and the lower limit of the confidence interval falls below the 0.7 threshold for a good model.

When the CIPHER model was used to predict maternal death alone, the AUROC was 0.87 (95% CI 0.86 to 0.88). The discriminative performance of the APACHE 2 score for death during pregnancy or less than 6 weeks post-partum in our ICU cohort was also high (AUROC 0.84, 95% CI 0.72 to 0.96). This analysis included only 433 women who had complete data on all 17 APACHE 2 predictor variables.

## Discussion

### Main findings

We have developed and internally validated the CIPHER clinical risk prediction model to accurately assess risk of either death or the need for life-saving prolonged organ support for pregnant or recently pregnant women admitted to an ICU at 13 international sites. The final CIPHER model includes predictor variables that are readily available globally and at relatively low cost. It is a simple model, including only 10 predictors. After internal validation, CIPHER affords high discrimination (0.82, 95% CI 0.81 to 0.84) and good calibration (slope of 0.92, 95% CI 0.91 to 0.92 and intercept of −0.11, 95% CI −0.13 to −0.08). External validation of the model is now required prior to implementation of the model in clinical practice.

### Strengths and limitations

We have built on the previously successful work of this collaborative team and developed the model on the basis of clinical knowledge and *a priori* information about relevant and globally available predictor variables. For this study, we chose to use a composite outcome that reflects the important health burden of severe maternal morbidities and goes beyond the traditional focus of ICU risk scores on death alone. We believe that this greatly expands the clinical utility of the CIPHER model. Maternal mortality is thankfully on the decline. As mortality declines, severe maternal morbidity will become even more relevant as an outcome to structure management strategies around. This model can now be easily recalibrated for individual settings as long as the 10 predictor variables are available.

The CIPHER model was developed specifically for use in obstetric ICU populations with a globally diverse cohort involving collaboration across 11 countries. Although inclusion of a diverse geographic sample leads to an increase in global relevance, it also likely contributed to a reduction in overall model performance at the local level. A reduction in overall performance is evident when we compare performance seen in the high- and low- or middle-income country subgroups. In both settings, performance is maintained above the threshold for an adequate prognostic model (AUROC >0.7) but CIPHER is better at discriminating between women with and without outcomes in the low- or middle-income population, where the majority of outcomes occurred. A potential weakness of the dataset used is the variability in outcome rates between sites; 88.6% of outcomes occurred in LMIC sites, 57.6% solely in the Pakistan site. For this reason, we recommend external validation and, if required, recalibration of the model in each setting individually before application in clinical care.

Performance of the CIPHER model to predict maternal death alone was similar to the APACHE II model in our cohort. We chose to use both maternal death and the need for life-saving organ support as a primary outcome in order to make the CIPHER model more clinically useful for a pregnant population than a model such as APACHE II, which predicts death alone. Severe maternal morbidity is as significant in its life-altering consequences as mortality within this young and otherwise healthy population of women.

Weaknesses of the study design included its retrospective nature, missing data due to incomplete data entry, and unmeasured variables from some sites, precluding the potential for inclusion of those variables in the final model. Over 100 cases were excluded because of a high degree of missing data that we felt would have undermined the benefit of the multiple imputation strategy to correct for missing data in the remaining cases. This resulted in a smaller dataset than had been targeted on the basis of our sample size calculation. We did not achieve our *a priori* estimated sample size, but use of multiple imputation in the remaining cases and the higher rate of adverse outcomes than had been expected resulted in an adequate sample size for model development. It is important to note that if we were to use the same sample size formula with our observed outcome rate of 16.5%, a sample size of 606 would be considered adequate to generate robust estimates of model coefficients. We far exceeded that with our sample size of 769 women in the cohort used for analysis.

Another limitation is that because clinicians were not masked to the results of the variables assessed in the modeling, CIPHER is vulnerable to treatment paradox [30]; this may be particularly true for the protective nature of post-surgical status that may modify both admission and intervention thresholds and surveillance intensity.

### Interpretation

Identifying the variables that predict outcome in pregnant or recently pregnant women admitted to the ICU and developing a prediction model enables estimation of the likelihood of an adverse maternal event in the future on the basis of information available at the time of a woman’s admission to the ICU. The candidate predictor variables for the CIPHER model were those that were routinely and reliably measured, were readily available in hospitals worldwide, and had potential to inform or predict severity of illness or outcome. The definition of severe maternal morbidity was organ- and management-based, reflecting the true burden of disease in the ICU: both the need for organ support and the impact of prolonged duration of organ support and care.

In the development cohort, a threshold CIPHER score of at least 50% was deemed a “positive” test for the combined outcome to define a high-risk group. The LR of 27.97 (95% CI 16.91 to 46.27) for this group is strongly informative. In this group, with an at least 50% CIPHER risk, there is evidence for action as it identifies those women who are most at risk of a combined outcome. In areas where resources are available to manage additional case load or where greater concern exists around impact of missing true-positive cases, setting the threshold for high risk as greater than 25% predicted probability remains informative with an LR of 9.18 (95% CI 6.16 to 13.69) with only a small increase in associated false-positive rate. This means that useful clinical information can be gained from the CIPHER model in order to guide care in a variety of contexts.

Four published studies have focused on development of a maternal ICU outcome prediction model [10, 31–33]. Developed solely in Brazil, the Maternal Severity Index used predefined, rather than statistically driven, WHO severity markers, identifying seven predictors of maternal death, many of which were themselves composite predictors [10]. A secondary analysis of a cohort of maternal general ICU admissions from the UK that focused on evaluation of APACHE II variables identified medical history, heart rate, systolic blood pressure, and especially Glasgow Coma Scale score as independently predictive of maternal death [31]; however, they did not develop a multivariable prediction model. Nine independent variables predictive of maternal death were identified in a West African (non-ICU) hospital-based study [32]. Of these, many were indications for ICU admission in our study and were not included as candidate predictor variables, including severe anemia, malaria diagnosed during pregnancy, obstetric hemorrhage, pre-eclampsia or eclampsia, uterine rupture, and genital infection or sepsis. Again, these variables were not formally combined to generate a comparable predictive model. A US military cohort was used to develop a 13-variable risk assessment model to predict 38 maternal outcomes, including labor, delivery, maternal morbidity, and death [33]. Performance of this model was good with a reported AUROC of 0.75 for poor maternal outcome, but the model is applicable only to the antenatal period. None of these studies resulted in models overtly applicable to critical care of obstetric patients in both HICs and LMICs.

Future work in this area of research should focus on a number of translational biomarkers poised to become regular components of both maternity and critical care and with potential to modify CIPHER [34, 35]. Prospective external and temporal validation studies of CIPHER are required prior to its broad dissemination into communities of care, whether in LMICs or HICs. To support work towards external validation we have provided a CIPHER calculator (see Additional file 2). An additional goal for validation could be to expand the scope of the CIPHER model to recalibrate in a population admitted to a high-risk maternity unit in order to guide decisions around admission to the ICU.

## Conclusions

The CIPHER model determines the risk of death or need for significant organ support in a population of pregnant and post-partum women receiving critical care, with clinical utility in both HICs and LMICs. CIPHER has the potential to be a pragmatic risk prediction tool to identify women at highest risk for adverse ICU outcomes and to assist with counseling patients and their families regarding management within the ICU. Ultimately, once validated, the CIPHER model could be applied globally to reduce the burden of pregnancy-related morbidity and mortality.

## Additional files

Additional file 1: **Table S1.** CIPHER (Collaborative Integrated Pregnancy High-dependency Estimate of Risk) cohort collaborators and site contribution. **Table S2.** Patient characteristics and univariate analysis results generated through complete case analysis. **Table S3.** Characteristics of women with and without missing data. (DOCX 43 kb)

Additional file 2: CIPHER (Collaborative Integrated Pregnancy High-dependency Estimate of Risk) model clinical calculator. (XLSX 12 kb)

## Abbreviations

ABG: Arterial blood gas
APACHE: Acute Physiology And Chronic Health Evaluation
aPTT: Activated partial thromboplastin time
AUROC: Area under the curve of the receiver operating characteristic
CI: Confidence interval
CIPHER: Collaborative Integrated Pregnancy High-dependency Estimate of Risk
HIC: High-income country
ICU: Intensive care unit
LASSO: Least Absolute Shrinkage and Selection Operator
LMIC: Low- and middle-income countries
LR: Likelihood ratio
SOFA: Sepsis-related Organ Failure Assessment
WHO: World Health Organization
