# Supplementary material for: Development and internal validation of the multivariable CIPHER (Collaborative Integrated Pregnancy High-dependency Estimate of Risk) clinical risk prediction model
Source: Crit Care. 2018 Oct 30;22:278. doi: 10.1186/s13054-018-2215-6 (PMC6206915; doi:10.1186/s13054-018-2215-6)
Supplement: Supplementary file 1 — Table S1. CIPHER (Collaborative Integrated Pregnancy High-dependency Estimate of Risk) cohort collaborators and site contribution. Table S2. Patient characteristics and univariate analysis results generated through complete case analysis. Table S3. Characteristics of women with and without missing data. (DOCX 43 kb) [file 13054_2018_2215_MOESM1_ESM.docx]

**Tables for CIPHER paper:**

Table S1: CIPHER cohort collaborators and site contribution

| **City, Country** | **Hospital name** | **Public / Private** | **Years data collected** | **Number outcome/population (Total=769)**  **n/N (%)** | **Obstetric Indication for ICU admission**  **n (%)** | **Antepartum Timing of Admission**  **n(%)** | **Maternal Age**  **(Years)** | **Parity**  **>=1**  **n(%)** |
| --- | --- | --- | --- | --- | --- | --- | --- | --- |
| **High Income** |  |  |  |  |  |  |  |  |
| Vancouver, Canada | St. Paul’s Hospital | Public, University | 2001-2011 | 0/57  (0%) | 42  (73.7%) | 15  (26.3%) | 36  [28-39] | 49 (86.0%) |
| Toronto, Canada | Mount Sinai Hospital | Public, University | 2005-2010 | 6/98  (6.1%) | 49  (50.0%) | 28  (28.6%) | 32  [28-36] | 86  (87.8%) |
| Melbourne, Australia | Monash Medical Centre | Public, University |  | 0/2  (0%) | 1  (50.0%) | 1 (50.0%) | [26-32] | 2  (100%) |
| Dublin, Ireland | The Rotunda Hospital and the National Maternity Hospital | Public, University | 2001-2012 | 5/74  (6.8%) | 40  (54.1%) | 9 (12.2%) | 33  [29-36] | 65  (87.8%) |
| New York, United States of America | Montefiore Medical Centre | Private, Community |  | 0/10  (0%) | 7  (70.0%) | 1 (10%) | 29  [27-32] | 9  (90%) |
| Amsterdam, Netherlands | AMC | Public, University |  | 4/36  (11.1%) | 28  (77.8%) | 10  (27.8%) | 31  [28-35] | 34  (94.4%) |
| **Middle Income** |  |  |  |  |  |  |  |  |
| Sao Paulo, Brazil | University of Campinas Hospital | Public, University |  | 0/12  (0%) | 9  (75.0%) | 4  (33.3%) | 26  [21-33] | 11  (91.7%) |
| Buenos Aires, Argentina | Hospital San Martin | Public, University |  | 33/142  (23.2%) | 98  (69.0%) | 38  (26.8%) | 26  [20-32] | 136  (95.8%) |
| Buenos Aires, Argentina | Sanatorio Anchorena | Private |  | 1/103  (1.0%) | 81  (78.6%) | 36  (35.0%) | 34  [29-37] | 93  (90.3%) |
| Malatya, Turkey | Inonu University Medical Faculty | Public, University |  | 2/20  (10.0%) | 17  (85.0%) | 0  (0%) | 32  [28-36] | 20  (100%) |
| Amman, Jordan | King Abdullah University Hospital | Public, University |  | 0/1  (0%) | 1  (100%) | 0  (0%) | n/a | 1  (100%) |
| Shanghai, China | Shanghai First Maternal and Fetal Care Hospital | Public, University |  | 0/15  (0%) | 13  (86.7%) | 0  (0%) | 33  [30-38] | 7  (46.7%) |
| Low Income |  |  |  |  |  |  |  |  |
| Karachi, Pakistan | Aga Khan University Health Centre | Private, University |  | 76/197  (38.6%) | 131 (66.5%) | 73  (37.1%) | 27  [25-32] | 189  (95.9%) |
| Total HIC |  |  |  | 15/277  (5.4%) | 167 (60.3%) | 64  (23.1%) | 33  [28-37] | 245  (88.4%) |
| Total LMIC |  |  |  | 112/492  (22.8%) | 350 (71.1%) | 151  (30.7%) | 29  [25-34] | 457  (92.9%) |
| Total |  |  |  | 769 | 127 |  |  |  |

Table S2: Patient characteristics and univariate analysis results generated through complete case analysis

| **Patient Characteristics** | **Descriptive statistic** | **Missing, n (%)** | **OR (95% CI)** | **p** | **Notes** |
| --- | --- | --- | --- | --- | --- |
| **Demographics** | | | | | |
| Age (years) | 30.3 ± 6.7 | 143 (16.3%) | 0.95 [0.92 – 0.98] | **<0.001** | APACHE IV |
| Marital status (married) | 448/821 (54.6%) married | 55 (6.3%) |  | | |
| BMI | 27.01 ± 5.01 | 453 (51.7%) |  | | |
| Prenatal care type |  | 102 (11.6%) |  | | |
| *Public Prenatal Care* | 489 (63.2%) |  |  |  |  |
| *Private Prenatal Care* | 285 (36.8%) |  |  |  |  |
| Gravidity | 3 [2 – 5] | 370 (42.2%) |  |  |  |
| Parity | 2 [1 – 3] | 404 (46.1%) |  |  |  |
| Smoking (no) | 497 (56.7%) | 328 (37.4%) |  |  |  |
| Gestational age at ICU admission (weeks) | 34.9 [28.8 – 38.9] | 456 (52.1%) |  | | |
| **Timing of ICU admission** | | | | | |
| External ICU Transfer (yes) | 228 (28.5%) | 75 (8.6%) | 2.14 [1.30 – 3.53] | **0.003** |  |
| Surgery in preceding 24 hours (yes) | 474 (56.6%) | 39 (4.5%) | 0.47 [0.32 – 0.70] | **<0.001** | APACHE IV |
| **Vital Signs** | | | | | |
| Temperature min (◦C) | 36.08 ± 0.79 | 280 (32.0%) |  |  | APACHE IV |
| Temperature max (◦C) | 37.47 ± 0.86 | 272 (31.1%) |  |  |  |
| Heart Rate max (BPM) | 119.8 ± 22.75 | 270 (30.8%) |  |  | APACHE IV |
| Heart Rate min (BPM) | 81.6 ± 17.9 | 270 (30.8%) |  |  |  |
| Respiratory rate min (BPM) | 25 [22 – 30] | 279 (31.8%) |  |  |  |
| SaO2 (%) | 96 [93 – 98] | 278 (31.7%) |  |  |  |
| Systolic Blood pressure max (mmHg) | 145 [129 – 161] | 193 (22.0%) | 0.99 [0.98 – 1.02] | **0.139** |  |
| Systolic Blood Pressure min (mmHg) | 100 [88 – 110] | 277 (31.6%) |  |  |  |
| Mean Arterial Pressure min | 73 [63 – 84] | 274 (31.3%) | 1.00 [0.98 – 1.01] | 0.560 |  |
| Mean Arterial Pressure max | 105 [93 – 119] | 197 (22.5%) | 0.99 [0.98 – 1.01] | 0.344 | SOFA |
| Shock Index Combined | 1.2 [0.9 – 1.5] | 263 (30.0%) | 1.85 [1.17 – 2.93 | **0.008** | calculated using min HR and max sBP or min HR and min sBP if max sBP missing |
| Diastolic max (mmHg) | 86 [75 – 98] | 214 (24.4%) | 1.00 [0.99 – 1.01] | 0.713 |  |
| Diastolic min (mmHg) | 59.5 [50 – 70] | 282 (32.2%) |  |  |  |
| Urine Output (g/24hr) | 2300 [1440 – 3440] | 286 (32.6%) |  |  | APACHE IV, SOFA |
| Glasgow Coma Score | 15 [9 – 15] | 175 (19.9%) | 0.85 [0.82 – 0.89] | **<0.001** | APACHE IV, SOFA |
| **Laboratory data** | | | | | |
| Haemoglobin (g/dL) | 8.6 [7.2 – 103] | 196 (22.4%) | 0.91 [0.84 – 0.99] | **0.026** |  |
| Haematocrit (%) | 26.9 [22.6 – 32.0] | 140 (16.0%) | 0.98 [0.95 – 1.00] | **0.069** | APACHE IV |
| White cell count (x10^9^/L) | 15.2 [11.4 – 21] | 107 (12.2%) | 1.04 [1.01 – 1.06] | **0.001** | APACHE IV |
| White cell count categorised | 600 (68.5%) high |  |  |  |  |
| Neutrophil | 22.9 [11.7 – 85.9] | 427 (48.7%) |  |  |  |
| Platelet count (x10^9^/L) | 131 [73 – 207] | 105 (12.0%) | 1.00 [1.00 – 1.00] | **0.153** | SOFA |
| Sodium | 137.97 ± 6.19 | 119 (13.6%) | 1.07 [1.04 – 1.10] | **<0.001** | APACHE IV |
| Urea | 6.88 [3.93 – 11.78] | 199 (22.7%) | 1.03 [1.01 – 1.04] | **0.001** | APACHE IV |
| Creatinine | 70.7 [53.1 – 113.2] | 113 (12.9%) | 1.00 [1.00 – 1.01] | **0.001** | APACHE IV, SOFA |
| Potassium | 3.93 ± 0.90 | 117 (13.4%) | 0.90 [0.72 – 1.12] | 0.338 |  |
| Bilirubin | 12.0 [6.8 – 27.0] | 232 (26.5%) | 1.01 [1.01 – 1.01] | **<0.001** | APACHE IV |
| Albumin | 23 [19 – 27] | 410 (46.8%) |  |  | APACHE IV, SOFA |
| AST | 34 [21 – 82] | 279 (31.8%) |  |  |  |
| ALT | 24 [13 – 65] | 171 (19.5%) | 1.00 [1.00 – 1.00] | <0.001 | not included in ICNARC |
| Lactate Dehydrogenase (LDH) | 498 [272 – 881] | 603 (68.8%) |  |  |  |
| PT | 12.3 [10.5 – 16.3] | 427 (48.7%) |  |  |  |
| INR | 1.15 [1 – 1.45] | 404 (46.1%) |  |  |  |
| APTT | 34 [29 – 43] | 244 (27.9%) | 1.02 [1.02 – 1.03] | <0.001 | not included in ICNARC |
| Microbiology Growth (yes) | 125 (24.8%) | 372 (42.5%) |  |  |  |
| Glucose | 5.8 [4.5 – 9.2] | 225 (25.7%) | 1.00 [0.99 – 1.00] | 0.384 | APACHE IV |
| Fibrinogen | 3.60 [2.15 – 6.25] | 654 (74.7%) |  |  |  |
| **ABG results** | | | | | |
| Highest FiO2 | 0.4 [0.25 – 0.5] | 451 (51.5%) |  |  | APACHE IV, SOFA |
| pH | 7.37 [7.30 – 7.44] | 220 (25.1%) | 0.68 [0.28 – 1.28] | 0.191 | APACHE IV |
| ABG SaO2 | 96.3 [92.2 – 98.8] | 398 (45.4%) |  |  |  |
| ABG Lactate | 2.5 [1.2 – 5.3] | 630 (71.9%) |  |  |  |
| **Perinatal Outcomes** | | | | | |
| Total Pregnancy Loss (yes) | 91 (12.9%) | 168 (19.2%) |  | | |
| *Spontaneous abortion* | 29 (31.9%) |  |  |  |  |
| *Induced abortion* | 62 (68.1%) |  |  |  |  |
| Stillborn | 65 (10.4%) | 252 (28.7%) |  |  |  |
| Liveborn | 559 (89.6%) | 252 (28.7%) |  |  |  |
| Birth Weight | 2500 [1670 – 3160] | 476 (54.3%) |  |  |  |

Table S3: Characteristics of women with and without missing data

|  | Missing (n=411)  Median (IQR) or n(%) | Complete (n=355)  Median (IQR) or n(%) |
| --- | --- | --- |
| Age (years) | 30 (25 – 35) | 31 (25 – 36) |
| BMI (kg/m^2^) | 27.0 (23.9 – 31.4) | 26.0 (23.5 – 29.1) |
| Gravidity | 3 (2 – 5) | 3 (2 - 4) |
| Parity | 1 (1 – 3) | 2 (1 – 3) |
| Systolic blood pressure (mmHg) | 143 (126 – 160) | 146 (130 – 163) |
| GCS | 15 (14 - 15) | 10 (5 – 15) |
| Platelets (x10^9^/L) | 144 (80 - 207) | 119.5 [ 64 - 207.5] |
| White blood cell count (x10^9^/L) | 14.3 (10.9 – 18.6) | 16.8 (12.5 – 23.4) |
| aPTT | 32.7 (28.4 - 40.2) | 35.0 (29.9 – 45.6) |
| Shock index | 1.16 (0.91 - 1.40) | 1.18 (1.00 - 1.52) |
| ICU length of stay (hours) | 51.9 (34.6 – 85.5) | 85.9 (48.0 - 156.4) |
| Creatinine (umol/L) | 70.7 (53.1 - 101.6) | 71.0 (53.1 - 132.6) |
| Sugery in previous 24hrs (y/n) | 240 (58.4%) | 183 (51.5%) |
| Primary Outcome (y/n) | 56 (13.6%) | 76 (21.4%) |
